# *Lentinus edodes* Exposure before and after Fetus Implantation: Materno-Fetal Development in Rats with Gestational Diabetes Mellitus

**DOI:** 10.3390/nu11112720

**Published:** 2019-11-09

**Authors:** Leticia F. Laurino, Fabia J. M. Viroel, Erika Caetano, Sara Spim, Thaisa B. Pickler, Raquel M. Rosa-Castro, Edilma Albuquerque Vasconcelos, Angela F. Jozala, Alessandre Hataka, Denise Grotto, Marli Gerenutti

**Affiliations:** 1 University of Sorocaba, Sorocaba-SP 18023-000, Brazil; leticia_fl_@hotmail.com (L.F.L.); fabia.viroel@prof.uniso.br (F.J.M.V.); erikacaetano01@hotmail.com (E.C.); sara.spim@edu.uniso.br (S.S.); thaisa.pickler@uniso.br (T.B.P.); raquel.rosa@prof.uniso.br (R.M.R.-C.); edilma.vasconcelos@prof.uniso.br (E.A.V.); angela.jozala@prof.uniso.br (A.F.J.); marli.gerenutti@gmail.com (M.G.); 2 Department of Veterinary Clinical Sciences, School of Veterinary Medicine and Animal Science, São Paulo State University (UNESP), Botucatu, São Paulo 18610-307, Brazil

**Keywords:** *Lentinus edodes*, mushroom nutraceuticals, gestational diabetes mellitus, anti-diabetic activity, pre-clinical study

## Abstract

Background: The presence of β-glucans and phenolic compounds in *Lentinus edodes* suggests this mushroom can be used as a nutritional supplement. Two gestational conditions (before and after fetus implantation) were evaluated, and *Lentinus edodes* exposure was performed in diabetes mellitus rat model induced by streptozotocin in pre-clinical tests. Methods: On the 20th day of pregnancy, cesarean sections were performed. Blood was collected for biochemical, hematologic parameters and oxidative stress biomarkers. Placenta and amniotic fluid were collected, and fetuses were analyzed through morphological evaluation. Results: The mushroom did not reduce the severe hyperglycemia of the mother-concept but promoted an increase in maternal insulin levels; reduced the levels of alanine aminotransferase, and aspartate aminotransferase, triglyceride and total cholesterol; protected the animals from post-implantation losses. Liver damage induced by streptozotocin was reversed in experimental groups. Conclusions: *Lentinus edodes* mushroom has antioxidant properties that can minimize the damage caused by gestational diabetes mellitus.

## 1. Introduction

Diabetes mellitus (DM) is a chronic disease that requires multifactorial strategies for glycemic control [1]. In diabetic patients, due to abnormal metabolism of insulin, cells and tissues do not use blood glucose, resulting in hyperglycemia, and these patients can develop other metabolic and functional complications [2].

Gestational diabetes mellitus (GDM) is a glucose intolerance and insulin resistance firstly detected during pregnancy. There is not an adequate treatment, effecting both mother and concept, increasing the risk of fetal loss, congenital malformations, premature birth and tendency to develop type 2 diabetes in the future [3,4,5]. Fetuses of diabetic mothers develop in a hyperglycemic intrauterine environment of oxidative stress. They adapt by altering insulin resistance and secretion, which impairs glucose tolerance in adult life and increases the risk of developing type 2 DM [6]. In addition, animal models have shown that diabetes can be transmitted to the fetus by intrauterine exposure to maternal hyperglycemia, which may contribute to a worldwide epidemic of diabetes, further emphasizing the need for adequate glycemic control during pregnancy [7,8].

Prevention and treatment of GDM are extremely important during the prenatal and postnatal phases. Physical activity and nutritional therapy are the first treatment choices for glycemic control, and insulin is only introduced when exercise and diet do not maintain normoglycemia [9].

The search for natural products with antidiabetic effects has increased in preventive medicine. Some species of edible mushrooms with high nutritional value and low-calorie content are recommended for diabetic patients due to the presence of phenolic compounds, which can reduce the oxidation of macromolecules [10,11].

*Lentinus edodes*, popularly known as shiitake, is one of the most consumed and studied mushroom species in the world, not only for its nutritional value but also for its potential therapeutic actions [12]. This mushroom contains essential nutrients such as proteins, carbohydrates, fatty acids (linoleic, palmitic and oleic), in addition to being used in low-calorie diets. The presence of tocopherols and phenolic compounds (*p*-hydroxybenzoic, *p*-coumaric and vanillic acid) indicates that this mushroom can be used as a supplement with high antioxidant power [13]. Regular consumption of this mushroom can increase immune function, improve the metabolic profile and reduce lipid levels and oxidative stress [14,15]. A previous study from our working group correlated the *Lentinus edodes* exposure with improvement in severe GDM [16]. Otherwise, we aimed to investigate the safety use of *Lentinus edodes* as a therapeutic aid in two GDM conditions: (i) exposure to *Lentinus edodes* from gestational day 1 to 19 (before fetus implantation) and (ii) exposure to *Lentinus edodes* from gestation day 9 to 19 (after fetus implantation), considering their potentialities as functional food. Fetal measurements, hematologic, metabolic and oxidative stress outcomes were examined.

## 2. Materials and Methods

### 2.1. Ethical Approval of the Study Protocol

The study was approved by the Commission of Ethics in the Usage of Animals of the University of Sorocaba (approval number 089/2016; São Paulo, Brazil). All experiments were carried out according to the international guideline—ARRIVE (Animal Research: Reporting of in Vivo Experiments) [17,18].

### 2.2. Lentinus edodes: Composition and Dose Selection

*Lentinus edodes* (Berk.) Pegler-cultivated strain H600 (Hokken, Shimotsuga-gun, Japan) was provided by the commercial company Yuki Mushrooms (São Paulo, Brazil). The samples of fresh mushroom were trimmed, cut into slices and lyophilized (Thermo Fisher Scientific, Waltham, MA, USA) for 48 h; the yield reached the ratio of 10% dry mass. The dried sample was milled (Marconi^®^ Piracicaba-SP, Brazil) and sieved (50 mesh and 60 mesh) and packed in airtight plastic containers.

Characterization included phenolic compounds (method adapted from Scalbert, Monties, Janin [19] and β-glucans quantification (β-Glucan Assay kit—Yeast and Mushroom; Megazyme, Bray, Ireland). The moisture content, total ashes, lipid and protein dosages were determined according to adapted methods described by Adolf Lutz Institute [20].

Daily doses of 100 mg/kg of *Lentinus edodes* was chosen based in Grotto et al. [21] study, in which the mushroom did not promote hepatic damage in rats.

### 2.3. Animal Experiments

Wistar rats (*Rattus norvegicus* var. *albinus*), which body weight varied from 250 to 300 g for the males and from 180 to 200 g for the females, were purchased from Biotério de Produção de Ratos, University of São Paulo, São Paulo State, Brazil). Animals were housed in Alesco^®^ (Monte Mor, SP, Brazil) microenvironment isolation cages under standard environmental conditions (23 °C, 12:12 h dark/light cycle). Industrialized dry food (Purina^®^, São Paulo, Brazil) and tap water ad libitum were provided.

### 2.4. Assays with Gestational Diabetes Mellitus Induced by Strepzotocina (GDM-STZ)

One male was housed up with four females for overnight periods, to mating. Each morning, the vaginal smear was taken under the microscope [22]. The presence of sperm (Biological Microscope, Axio Lab. A1, ZEISS, São Paulo, Brazil), was designated as gestational day 1. On the eighth day of pregnancy, fasting blood glucose (6 hours) was dosed in the Freedom Lite Freestyle (Abbott). Diabetes was induced by administration of streptozotocin (40 mg/kg) diluted in 0.1 mol/L citrate buffer (pH 4.5) intravenously. After 48 hours, the fasting glucose (6 hours) was performed again. Animals with glycemia above 120 mg/dL were considered diabetic [23,24].

Twenty-four pregnant females caged in isolation and they were randomly assigned to one of four groups:

(I) Negative control (SC: saline control 0.9% from day 1 to day 19 of the gestational period);

(II) Positive control (GDM + S: diabetic + saline solution 0.9% from day 1 to day 19 of the gestational period);

(III) *Lentinus edodes* (Le) before (b) GDM-STZ: GDM + Leb;

(IV) *Lentinus edodes* (Le) after (a) GDM-STZ: GDM + Lea.

The condition “before” means 100 mg/kg/day *Lentinus edodes* from gestation day 1 to 19 (before fetus implantation) and “after” means 100 mg/kg/day *Lentinus edodes* from gestation day 9 to 19 (after fetus implantation).

### 2.5. Oral Glucose Tolerance Test (OGTT)

Oral glucose tolerance tests (OGTT) were conducted at day 17 of pregnancy [25]. Briefly, after fasting for 6 h, the rats received 2 g of dextrose/kg body weight and blood was collected at 0, 10, 20, 30, 60 and 120 minutes, using Glycemic Monitor FreeStyle Lite, Abbott.

### 2.6. Blood Collection and Reproductive Performance

On the 20th day of pregnancy, females were anesthetized with xylazine (6 mg/kg) and ketamine (100 mg/kg) administered by intraperitoneal injection. Cesarean sections were performed using a longitudinal incision along the linea alba. Blood samples were collected from hepatic veins and divided into two tubes. One tube containing ethylenediamine tetra-acetic acid (EDTA) anticoagulant, for blood cell counts and oxidative stress evaluation. One tube with gel separator for biochemical evaluation. Females were then euthanized and their womb and ovaries were removed. All sampled materials were maintained at −80 °C until analysis. Uterine cavities were checked for the number of fetuses, implantations and visible resorptions. The ovaries and uterus were weighed, and corpus luteal counted.

### 2.7. Maternal Hematologic Parameters

Blood was analyzed in a Sysmex^®^ XS-1000i (São José dos Pinhais, PR, Brazil) apparatus to determine: red blood cell (RBC), hemoglobin, hematocrit, platelet (PLT) and white blood cells (WBC) counts.

### 2.8. Maternal Biochemical Profile

The biochemical parameters were measured in an automated system (Cobas 111 Roche^®^ spectrophotometer, São Paulo, Brazil). The following parameters were determined: alanine aminotransferase (ALT), aspartate aminotransferase (AST), albumin, creatinine, urea, glucose, triglycerides, total cholesterol and high-density lipoprotein cholesterol (HDL-Chol).

### 2.9. Embryofetal Development and Placental Analysis

Amniotic fluid was collected from gestational sacs and the samples were stored immediately at −80 °C. Plasma and amniotic insulin dosages were quantified by the Elisa solid phase test, based on the Sandwich principle, using a commercial kit (Rat/Mouse Insulin Elisa Kit–Merck^®^).

Morphological effects were checked comparing body measurements. Length in mm of the sections: anteroposterior and latero-lateral of the skull, anteroposterior and latero-lateral of the thorax, cranium-caudal and tail were measured using a pachymeter.

Placentas were weighed and stored immediately after collection at −80 °C. Oxidative stress biomarkers were performed in a homogenate made with 250 mg of pooled placentas and 5 mL 1.15% KCl. The tissue was homogenized using an ultrasonic processor, always in an ice bath.

### 2.10. Oxidative Stress Biomarkers

Thiobarbituric acid reactive substances (TBARS), reduced glutathione (GSH), glutathione peroxidase (GPx) and catalase (CAT) were evaluated in both blood and placenta homogenate.

Enzyme CAT activity was evaluated by UV/VIS spectrophotometry following Aebi [26] method. For placenta, 20 µL homogenate was diluted in 1910 μL phosphate buffer and H_2_O_2_ was added, initiating the reaction. The activity of the enzyme was calculated based on the fresh placenta mass (0.250 g). To the blood, a constant of variation (k), related to hemoglobin (Hb), was used to obtain CAT blood activity (k/g Hb).

To determine the antioxidant levels of GPx, Paglia and Valentine [27] method were followed, based on the oxidation of the reduced form of nicotinamide adenine dinucleotide phosphate (NADPH).

For GSH, Ellman [28] method was followed for the quantification of total reduced thiols. A calibration curve with predefined concentrations of GSH (0.005, 0.01, 0.025, 0.05 and 0.1 mM) was employed.

TBARS were quantified using Ohkawa; Ohishi; Yagi [29] method with modifications. Briefly, A calibration curve with different concentrations of malondialdehyde (MDA) standard was used to calculate the concentration of MDA in plasma.

### 2.11. Statistical Analyses

Data are presented as mean ± SD (standard deviation; *n* = 6/group) and the homoscedasticity was evaluated by Bartlett test. Homogeneous data were analyzed by ANOVA and Tukey–Kramer test in multiple posterior comparisons. Chi-square test was used evaluate differences in pre-implantation loss and *p*-values < 0.05 were considered significant. Results were analyzed using GraphPad Prism Software (San Diego, CA, USA)

## 3. Results

### 3.1. Lentinus edodes

*Lentinus edodes* presented 39 g of beta-glucans per 100 g of dry mushroom and 1.48 mg of GAE/g of phenolic compounds. In addition, this mushroom presented: moisture (7.3%–7.5%), minerals (5.4%–5.8%), lipids (2.3%–3.1%) and proteins (20.3%–21.5%).

### 3.2. Gestational Evaluation

After 48 hours of streptozotocin administration, all GDM groups showed increased glycemic levels compared to the SC (SC = 60.0 ± 5.0, GDM + S = 202.3 ± 34.7, GDM + Leb = 209.3 ± 36.0, GDM + Lea = 193.0 ± 22.7 mg/dL—F = 19.26, *p* < 0.001), showing the development of GDM.

On the 20th day of pregnancy, females were weighed and the mass gain was calculated. Animals from GDM groups showed a reduction in the mass gain compared to SS group (SC = 110.3 ± 26.8, GDM + S = 59.0 ± 16.0, GDM + Leb = 54.0 ± 11.0, GDM + Lea = 54.0 ± 29.0 g—F = 3.919, *p* = 0.0027).

### 3.3. Glycemic Profile on GMD

**Figure 1:**
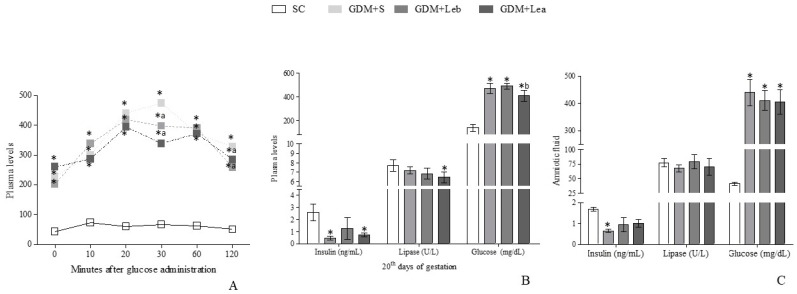
Pancreatic endocrine and exocrine functions tests. (**A**) Oral glucose tolerance test (OGTT) on 17th day of gestation. (**B**) Serum concentration of insulin, lipase and glucose. And Leb is *Lentinus edodes* before fetus implantation) b is before. (**C**) Amniotic fluid concentration of insulin, lipase and glucose. SC (saline control 0.9%), GDM + S (diabetic + saline solution 0.9%), GDM + Leb (diabetic + 100 mg/kg/day *Lentinus edodes* before fetus implantation) and GDM + Lea (diabetic + 100 mg/kg/day *Lentinus edodes* after fetus implantation). Data are presented as mean ± SD (*n* = 5). * *p* < 0.05 in comparison to SC group, a in comparison to GDM + S group, one-way ANOVA, followed by Tukey–Kramer’s.

Pancreatic endocrine and exocrine functions tests are reported in Figure 1. The oral glucose tolerance test (OGTT) showed differences among all GDM groups and SC (F = 19.26, *p* < 0.001) in total studied times and reduction of the glycemic profile in GDM + Leb and GDM + Lea animals in relation to GDM + S after 30 and 120 minutes (F = 19.26, *p* < 0.001; F = 19.26, *p* < 0.05, respectively).

Insulin levels in GDM + S group in both plasma and amniotic fluid were reduced in relation to SC (F = 7.241, *p* = 0.0059; F = 4.332, *p* = 0.0205, respectively) and in GDM + Lea group the plasmatic concentration remained reduced compared to the SC. In the same way, only plasmatic lipase’s concentration in GDM + Lea group presented a significant reduction when compared to the SC (F = 4.672, *p* = 0.0139). Glucose levels in both plasma and amniotic fluid were higher in GDM groups than SC. After 20 days, a persistent and severe GDM was observed.

### 3.4. Maternal Hematologic and Biochemical Evaluation

**Figure 2:**
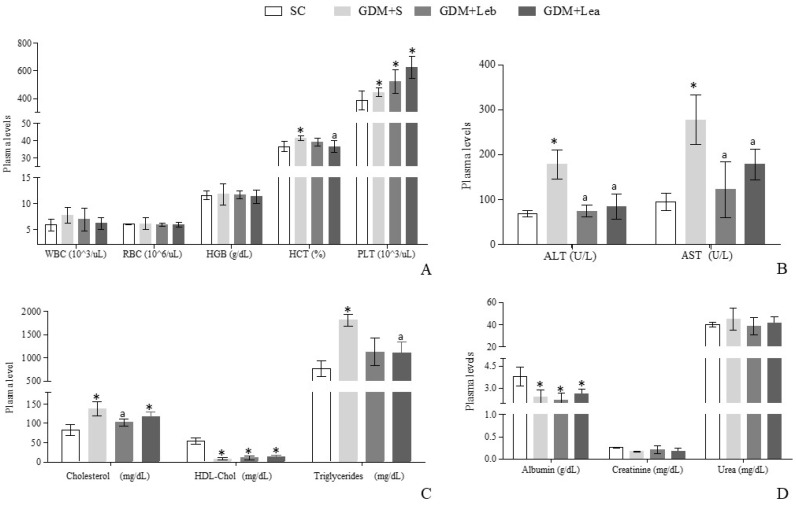
Hematological, hepatic, renal and lipid profiles outcomes of pregnant rats. (**A**) Hematological parameters. (**B**) Biochemical serum outcomes for hepatic analysis. (**C**) Lipidic profile. (**D**) Biochemical serum outcomes for renal analysis. SC (saline control 0.9%), GDM + S (diabetic + saline solution 0.9%), GDM + Leb (diabetic + 100 mg/kg/day *Lentinus edodes* before fetus implantation) and GDM + Lea (diabetic + 100 mg/kg/day *Lentinus edodes* after fetus implantation). WBC (leukocyte), RBC (red blood cells), HGB (hemoglobin), HCT (hematocrit) and PLT (platelet). ALT (alanine aminotransferase) and AST (aspartate aminotransferase). Data are presented as mean ± SD (*n* = 6) or percentage. * *p* < 0.05 in comparison to SC group, a in comparison to GDM + S group, one-way ANOVA, followed by Tukey–Kramer’s.

Figure 2 shows hematological, hepatic, renal and lipid profiles analyzes. Regarding hematological parameters, there was an increase in platelet levels (F = 3.674, *p* = 0.0491) in all GDM groups compared to SC group. There was an increase of hematocrit in GDM + S when compared to SC group, however GDM + Lea presented reduction in the hematocrit in relation to GDM + S group (F = 3.965, *p* = 0.0329).

Hepatic cell injury was observed with the increase in AST (F = 9.968, *p* = 0.0014) and ALT levels (F = 23.23, *p* < 0.0001) in GDM + S group compared to the other groups. All groups presented the De Ritis ratio rate greater than 1. Regarding renal function, there was a decrease in the albumin parameter (F = 5,189, *p* = 0.0100) in all GDM animals in relation to the SC. Creatinine (F = 1.349, *p* = 0.2917) and urea levels (F = 0.8366, *p* = 0.4923) were not altered.

All GDM rats showed a reduction in HDL-chol levels in relation to the SC group (F = 53.63, *p* < 0.00001). GDM + S and GDM + Lea groups showed an increase in total cholesterol levels (F = 9.973, *p* = 0.0018) compared to the SC group. However, GDM + Leb showed a reduction in total cholesterol when compared to GDM + S. There was an increase in triglycerides levels (F = 4.417, *p* = 0.0413) in GDM + S in relation to the SC group, and a reduction of this parameter in GDM + Lea in relation to GDM + S.

### 3.5. Reproductive Performance of Female Rats

The animal’s reproductive capacity is shown in Table 1. It can be observed a reduction in uterus weight in GDM + S group in relation to SC group (F = 0.5187, *p* = 0.6774). The ovaries weight decreased (F = 3.336, *p* = 0.0287) and placental weight increased (F = 10.66, *p* < 0.0001) in GDM + S and GDM + Leb groups compared to the SC group. An increase in post-implantation losses was observed in GDM + S and GDM + Lea groups compared to the SC (*p* = 0.0287), and GDM + Leb presented a reduction in the same parameter when compared to GDM + S. No changes were observed in the number of live fetuses per mother among GDM groups and SC (F = 0.8614, *p* = 0.4790).

### 3.6. Embriofetal Development

**Figure 3:**
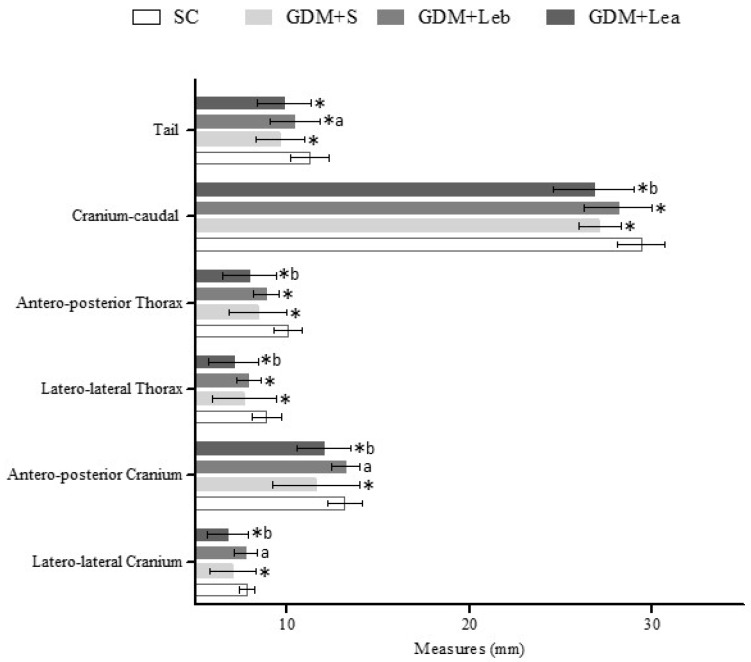
Mean length (mm) of sections of the head and body of fetuses. SC (saline control 0.9%), GDM + S (diabetic + saline solution 0.9%), GDM + Leb (diabetic + 100 mg/kg/day of *Lentinus edodes* before fetus implantation) and GDM + Lea (diabetic + 100 mg/kg/day of *Lentinus edodes* after fetus implantation). Data are presented as mean ± SD or percentage. * *p* < 0.05 in comparison to SC group, a in comparison to GDM + S group, one-way ANOVA, b in comparison to GDM + Leb, followed by Tukey–Kramer’s.

Figure 3 is presenting the mean length (mm) of sections of the head and body of fetuses. GDM + S presented reduction in all morphometric measures when compared to SC. Otherwise, GDM + Leb group presented increase in cranium (F = 14.51, *p* < 0.001) and tail (F = 12.14, *p* < 0.001) measurements when compared to GDM + S group and reduction in thorax (F = 12.04, *p* < 0.001), cranium–caudal (F = 15.64, *p* < 0.001) and tail (F = 13.52, *p* < 0.001) measurements when compared to SC group. GDM + Lea group presented a reduction of the skull, thorax and cranium–caudal measurements (F = 16.28, *p* < 0.0001) when compared to GDM + Leb group and also presented reduction of all parameters when compared to SC (Figure 3).

Regarding fetal weight, the data found were 2.19 ± 0.28 g for the SC group, 1.93 ± 0.16 g for GDM + S, 2.04 ± 0.24 g for GDM + Leb and 1.83 ± 0.35 g for GDM + Lea. GDM + S fetus had a significant decrease in body weight compared do SC group (*p* < 0.05) as well as fetus from GDM + Lea mothers.

### 3.7. Oxidative Stress on Maternal Blood and Placenta

**Figure 4:**
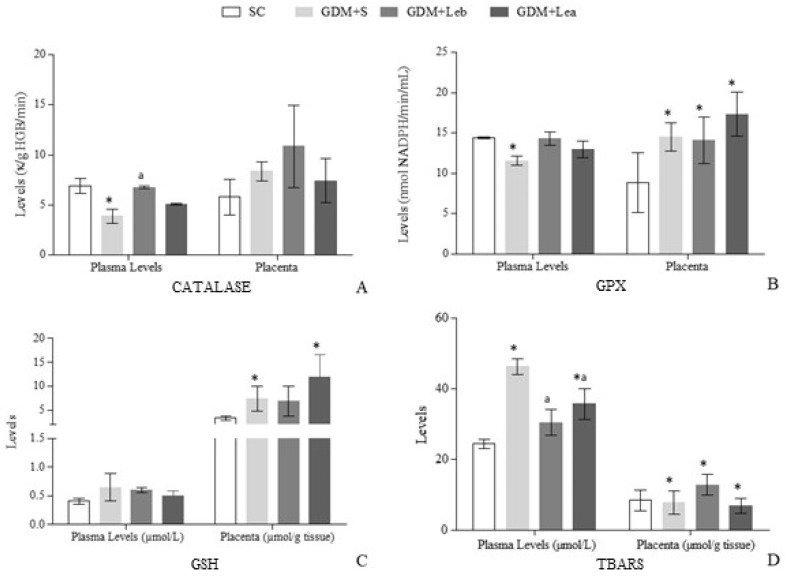
Activity level of catalase, reduced glutathione (GSH), glutathione peroxidase (GPx) and thiobarbituric acid reactive substances (TBARS) in blood and placenta of pregnant rats. (**A**) Catalase activity in total blood and placenta. (**B**) GPx activity in total blood and placenta. (**C**) GSH concentration in total blood and placenta. (**D**) TBARS concentration in plasma and placenta. SC (saline control 0.9%), GDM + S (diabetic + saline solution 0.9%), GDM + Leb (diabetic + 100 mg/kg/day of *Lentinus edodes* before fetus implantation) and GDM + Lea (diabetic + 100 mg/kg/day of *Lentinus edodes* after fetus implantation). Data are presented as mean ± SD or percentage. * *p* < 0.05 in comparison to SC group, a in comparison to GDM + S group, one-way ANOVA, followed by Tukey–Kramer’s.

The oxidative stress results are shown in Figure 4. There was a decrease in blood CAT activity (F = 4.788, *p* = 0.0156) in GDM + S in relation to the SC, and in GDM + Leb this parameter increased in relation to GDM + S. CAT activity in placenta did not present significant alterations. Regarding GPx in total blood, GDM + Leb and GDM + Lea groups presented no difference compared to the SC. On the other hand, GPx activity presented a reduction in GDM + S group in relation to the SC group (F = 3.212, *p* = 0.0617). All GDM groups presented increased GPx placental activity in relation to the SC (F = 7.811, *p* = 0.0015).

There were no differences in GSH levels in blood. In placenta, there was an increase in GSH levels in GDM + S and GDM + Lea in relation to the SC (F = 5.871, *p* = 0.0056). Regarding TBARS in plasma, an increase was observed in GDM + S and in GDM + Lea (F = 9,791, *p* = 0.0012) compared to the SC. However, TBARS levels decreased in GDM + Leb and GDM + Lea in relation to GDM + S. In placenta, TBARS concentration increased in GDM + Leb and decreased in GDM + S and GDM + Lea in relation to the SC (F = 5.150, *p* = 0.0096).

## 4. Discussion

In this study, the safety use of *Lentinus edodes* was evaluated in GDM before fetus implantation and after fetus implantation through fetal measurements, hematologic, metabolic and oxidative stress outcomes. *Lentinus edodes* did not reduce the mother hyperglycemia, however it promoted an improvement in maternal glucose tolerance and an increase in insulin levels. *Lentinus edodes* protected the animals from the embryo post-implantation losses when administered before GDM-STZ. The mushroom intake was safe, had antioxidant properties and reversed the liver damage caused by STZ.

STZ is an antimicrobial agent used for decades due to its well-characterized diabetogenic effect as a pancreatic β-cytotoxic drug [30]. GDM-STZ is a well-established experimental model for the evaluation of insulin deficiency and hyperglycemic effects on fetuses [31]. However, Caluwaerts et al. [32] affirm that only doses higher than 40 mg/kg STZ are a good model of GDM-STZ studies. On the other hand, the great variability of plasma glucose levels allows defining GDM-STZ as moderate (glycemia between 120 and 200 mg/dL) or severe (glycemia superior to 200 mg/dL). In our trials, although the dose of STZ was considered low, the animals developed severe GDM-STZ.

The glucose oxidation and the glycation of non-enzymatic proteins, associated with the increase of oxygen free radicals, favor the development of diabetic complications in pregnant women. Therefore, exogenous antioxidant agents such as functional foods are useful to prevent and treat DM-STZ [33,34]. *Lentinus edodes* mushroom, a promising functional food, has essential nutrients being used in low-calorie diets and in the prevention of diseases related to oxidative stress [13].

Studies with GDM-STZ have shown that pregnant diabetic rats present a reduction in weight gain when compared to non-diabetic animals [35,36], likewise our findings. However, this reduction in weight gain was not reversed by *Lentinus edodes*.

In SC rats, insulin absorption in OGTT was in agreement to the physiological response and blood glucose levels returned to the original state at the end of the test [37]. However, in diabetic rats, there were no reduction in response time or blood glucose levels, which were increased in all studied periods when compared to the control group [38,39]. Intrauterine environment in moderate diabetes is associated with deficiencies in insulin secretion in adult life, while pups born from mothers with severe diabetes present depleted insulin action in adult life [7]. In groups treated with *Lentinus edodes*, there was a reduction in response time or blood glucose levels.

Regarding glycemic profile, *Lentinus edodes*, even without reducing the severe hyperglycemia, improved maternal glucose tolerance in both maternal and amniotic fluid when administered before STZ. Zhang et al. [40] showed some compounds of *Lentinus edodes* are able to prevent the inhibition of insulin synthesis in DM-STZ, which could explain our results. On the other hand, pancreatic lipase showed STZ did not promote other pancreatic injuries in GDM rats, with preserved exocrine function. Regarding endocrine function, β-pancreatic cells were damaged possibly due to the route and dose used for STZ administration. However, pancreas was better preserved in the group exposed to the mushroom since the first day (GDM + Leb). When blood insulin levels are high, glucose should be lower; contrary, GDM + Leb group had higher insulin levels and blood glucose levels remained increased, indicating, possibly, insulin resistance.

DM-STZ can induce important hematological alterations in both white and red blood cells [41,42]. DM-STZ can also increase the number of platelets, inducing an increase in platelet aggregation [43]. Normal levels of urea and creatinine and elevated platelet levels may be indicative of nephrotic syndrome secondary to diabetes, which consists of hypoalbuminemia associated with dyslipidemia [44,45]. In our study, *Lentinus edodes* maintained the high platelets counting in GDM-STZ animals. Cho, Mooney and Cho [46] showed that hyperglycemia in DM increases hematocrit values because it renders the erythrocyte membrane more rigid with the aggregation of red cells, causing an increase in viscosity. This elevation in hematocrit can also be explained by the increased permeability of the capillary wall. GDM groups treated with *Lentinus edodes* presented reduction of hematocrit.

Hepatic and renal abnormalities and dyslipidemia are common in GDM-STZ [47]. Elevated ALT and AST levels in blood represent injury liver cells and were observed in mice with DM-STZ [48,49]. The membrane permeability of liver cells may be dysfunctional due to the accumulation of fat in hepatocytes caused by insulin deficiency [50]. GDM + S group had higher ALT and AST levels compared to non-diabetic animals, but the diabetic groups treated with *Lentinus edodes* showed a decrease in ALT and AST levels when compared to the GDM + S group, indicating STZ damage was reversed by the mushroom. Akamatsu et al. [51] observed that the polyphenols from aqueous fractions of *Lentinus edodes* have hepatoprotective effects, decreasing the activity of ALT and AST in hepatic lesions induced by dimethylnitrosamine, reducing liver inflammation and cell death by necrosis. De Ritis ratio is calculated by dividing AST by ALT levels, and this ratio allows to observe the damage extension caused by toxic hepatitis [52]. All groups in this study had a De Ritis ratio greater than or equal to 1, which means the liver damage was severe but less extensive.

GDM + S group presented a lipid profile similar to those found in Afiune et al. [53] and El-Sayyad et al. [54] studies, confirming the reproducibility of GDM induction by STZ, in which GDM-STZ rats presented increased levels of triglycerides and cholesterol and reduced HDL-chol. Spim et al. [55], when evaluating the ingestion of *Lentinus edodes* associated with a high-fat diet, proved that this specie of mushroom has a hypocholesterolemic action, reducing total cholesterol and triglycerides levels. Xu et al. [56], when administering polysaccharides from *Lentinus edodes*, also observed a reduction in oxidative stress induced by hypercaloric diet in rats, in addition to the reduction of total cholesterol, triglycerides and low-density lipoprotein cholesterol (LDL-chol) levels. These results corroborate with Yu et al. [14] that observed the consumption of aqueous extract of Shiitake was able to reduce the levels of triglycerides. In our study, although *Lentinus edodes* was used as a food, it was also able to reduce triglyceride levels in GDM + Leb and GDM + Lea rats and, in addition, GDM + Leb group also showed a reduction in total cholesterol levels, when compared to GDM + S group.

In reproductive toxicity studies, special attention should be given to the placenta, whose main function is to act as an interface between the fetus and the mother [57]. An increase in placenta weight in GDM-STZ can be a compensation mechanism to maximize poor maternal-fetal nutrients exchange [36]. The increased placenta weight in GDM groups, treated or not with *Lentinus edodes*, was inefficient to reverse the low weight of the concepts at least in mothers exposed to the mushroom after fetus implantation.

Calderon et al. [58] and Saito et al. [59] correlated moderate maternal hyperglycemia with fetal macrosomia. They reinforced that maternal hyperglycemia alters the maturation of placentas and their irrigation. On the other hand, the authors also characterized the placentas from diabetic mothers as insufficiently nutritious, which is responsible for small fetuses. In a literature review, authors reported macrosomia could affect from 15% to 45% of babies born to diabetic mothers [60]. In our study, fetus did not have macrosomia; contrarily, fetuses from GDM mothers presented reduction of external morphological measures. These results are coherent to severe GDM-STZ. GDM + Leb promoted an increase in skull and tail parameters when compared to GDM + S, indicating a protective effect of the mushroom when administered before STZ. Moreover, *Lentinus edodes* mushroom was able to protect the animals from the post-implantation losses of the embryo when administered before the GDM-STZ induction.

Severe hyperglycemia during pregnancy and consequent fetal hyperglycemia, responsible for fetal growth retardation, may also be related to oxidative stress and changes in the intrauterine environment [61]. The high rate of glucose in the amniotic fluid can prolonged the fetuses β-pancreatic cells stimulation, causing a pancreatic insulin depletion and hypoinsulinemia [39]. Our results indicate that *Lentinus edodes* were able to revert fetal hypoinsulinemia in amniotic fluid, even though it did not reverse hyperglycemia.

Increased blood glucose is the main cause of oxidative stress in diabetes. STZ stimulates the generation of H_2_O_2_ in β-pancreatic cells that are destroyed by the production of nitrogen monoxide (NO) [62]. Moreover, oxidative stress occurs even in normal pregnancies due to the high metabolic activity of the placenta and maternal metabolism [63]. In GDM-STZ, reactive oxygen substances overproduction leads to the molecular and structural cell damage, whether in membranes, DNA, lipids or proteins [64,65].

Hyperglycemia can increase H_2_O_2_ production and deregulate the expression of catalase [66]. There is a decrease in its activity in different tissues and organs in DM [67], and the same happened in our study. However, administration of *Lentinus edodes* since the first day of gestation (GDM + Leb) increased catalase activity compared to GDM + S.

The antioxidant system of glutathione enzymes plays an important role in cell defense against reactive free radicals and other oxidant species [68]. If the glutathione system is altered, the cells are susceptible to oxidative stress and cellular injury [69]. GDM + S group presented a reduction in GPx activity compared to the SC; the groups treated with mushroom, although there was no significant difference, showed a tendency to approach the SC group. Our results are in accordance to Yurkiv et al. [70], who studied the influence of *Agaricus blazei* and *Ganoderma lucidum* mushrooms on the oxidative stress in rats with DM. They indicated the protective effect from antioxidant compounds of these mushrooms and also of *Lentinus edodes* and the reduction of toxic substances (such as H_2_O_2_). However, in placenta, GDM groups presented higher GSH level and GPx activity.

GDM-STZ intensified the production of free radicals and increases the lipid peroxidation by increasing TBARS levels. To prevent hydroxyl radicals formation can be a way of reducing damage [33], and *Lentinus edodes* mushroom presented an antioxidant potential since both treated diabetic groups showed a reduction of plasma TBARS concentration in relation to GDM + S.

## 5. Conclusions

The dose of 40 mg/kg of STZ induced severe and prolonged gestational diabetes. Although *Lentinus edodes* did not reduce the mother-fetus hyperglycemia, it promoted an improvement in maternal glucose tolerance and an increase in insulin levels. *Lentinus edodes* was unable to alter the reduction of maternal weight gain; however, it protected the animals from the post-implantation losses of the embryo when administered before GDM-STZ. The mushroom reversed the liver damage caused by STZ and reduced the triglycerides and cholesterol levels. *Lentinus edodes* mushroom has antioxidant properties that protected against the damage caused by hyperglycemia in GDM. The best protective effects both in mothers and in fetuses were observed when administered since the first day of gestation.

## Figures and Tables

**Table 1:** Reproductive capacity of female rats.

Groups	Uterus Weight (g)	Ovary Weight (g)	Placenta Weight (g)	Number of Alive Fetus Per Mother	Post-Implantation Loss (%)
SC	44.48 ± 13.83	0.142 ± 0.015	0.415 ± 0.09	9.75 ± 1.83	4.6
GDM + S	35.40 ± 12.49 *	0.112 ± 0.019 *	0.494 ± 0.143 *	8.16 ± 1.53	16 *
GDM + Leb	41.64 ± 8.36	0.105 ± 0.020 *	0.485 ± 0.102 *	10.83 ± 2.03	6.9 ^a^
GDM + Lea	42.79 ± 11.79	0.123 ± 0.019	0.443 ± 0.081	11.33 ± 2.92	13.8 *^b^
	(F = 0.5187, *p* = 0.6774)	(F = 3.336, *p* = 0.0287)	(F = 10.66, *p* < 0.0001)	(F = 0.8614, *p* = 0.4790)	(*p* = 0.0287)

SC (saline control 0.9%), GDM + S (diabetic + saline solution 0.9%), GDM + Leb (diabetic + 100 mg/kg/day *Lentinus edodes* before fetus implantation) and GDM + Lea (diabetic + 100 mg/kg/day *Lentinus edodes* after fetus implantation). Data are presented as mean ± SD or percentage. * *p* < 0.05 in comparison to SC group, a in comparison to GDM + S group, b comparison between groups exposed to the same mushroom, one-way ANOVA, followed by Tukey–Kramer’s or Chi-square test.

## References

[1] American Diabetes Association (ADA) (2017). American Diabetes Association standard of medical care in diabetes—2017. Diabetes Care.

[2] Lo H.-C., Wasser S.P. (2011). Medicinal mushrooms for glycemic control in diabetes mellitus: History, current status, future perspectives, and unsolved problems (review). Int. J. Med. Mushrooms.

[3] Wendland E.M., Torloni M.R., Falavigna M., Trujillo J., Dode M.A., Campos M.A., Duncan B.B., Schmidt M.I. (2012). Gestational diabetes and pregnancy outcomes—A systematic review of the World Health Organization (WHO) and the International Association of Diabetes in Pregnancy Study Groups (IADPSG) diagnostic criteria. BMC Pregnancy Childbirth.

[4] World Health Organization (WHO) (2016). World Health Organization Global Report on Diabetes.

[5] Coustan D.R. (2013). Gestational diabetes mellitus. Clin. Chem..

[6] Hanafi M.Y., Abdelkhalek T.M., Saad M.I., Saleh M.M., Haiba M.M., Kamel M.A. (2016). Diabetes-Induced perturbations are subject to intergenerational transmission through maternal line. J. Physiol. Biochem..

[7] Fetita L.S., Sobngwi E., Serradas P., Calvo F., Gautier J.F. (2006). Review: Consequences of fetal exposure to maternal diabetes in offspring. J. Clin. Endocrinol. Metab..

[8] Bueno A., Sinzato Y.K., Sudano M.J., Alvarenga Fda C., Calderon Ide M., Rudge M.V., Damasceno D.C. (2014). Short and long-term repercussions of the experimental diabetes in embryofetal development. Diabetes. Metab. Res. Rev..

[9] Wang C., Yang H. (2016). Diagnosis, prevention and management of gestational diabetes mellitus. Chronic Dis. Transl. Med..

[10] Wu T., Xu B. (2015). Antidiabetic and antioxidant activities of eight medicinal mushroom species from China. Int. J. Med. Mushrooms.

[11] Chandra L.C., Smith B.J., Clarke S.L., Marlow D., D’Offay J.M., Kuvibidila S.R. (2011). Differential effects of shiitake- and white button mushroom-supplemented diets on hepatic steatosis in C57BL/6 mice. Food Chem. Toxicol..

[12] Bisen P.S., Baghel R.K., Sanodiya B.S., Thakur G.S., Prasad G.B.K.S. (2010). *Lentinus edodes*: A Macrofungus with pharmacological activities. Curr. Med. Chem..

[13] Carneiro A.A.J., Ferreira I.C.F.R., Dueñas M., Barros L., Da Silva R., Gomes E., Santos-Buelga C. (2013). Chemical composition and antioxidant activity of dried powder formulations of *Agaricus blazei* and *Lentinus edodes*. Food Chem..

[14] Yu S., Wu X., Ferguson M., Simmen R.C., Cleves M.A., Simmen F.A., Fang N. (2016). Diets containing shiitake mushroom reduce serum lipids and serum lipophilic antioxidant capacity in rats. J. Nutr..

[15] Dai X., Stanilka J.M., Rowe C.A., Esteves E.A., Nieves C., Spaiser S.J., Christman M.C., Langkamp-Henken B., Percival S.S. (2015). Consuming *Lentinula edodes* (shiitake) mushrooms daily improves human immunity: A randomized dietary intervention in healthy young adults. J. Am. Coll. Nutr..

[16] Maschio B.H., Gentil B.C., Caetano E.L.A., Rodrigues L.S., Laurino L.F., Spim S.R.V., Jozala A.F., Santos C.A., Grotto D., Gerenutti M. (2017). Effects characterization of shiitake culinary-medicinal mushroom, *Lentinus edodes* (*Agaricomycetes*), on severe gestational diabetes mellitus in rats. Int. J. Med. Mushroom.

[17] Kilkenny C., Browne W.J., Cuthill I.C., Emerson M., Altman D.G. (2010). Improving bioscience research reporting: The ARRIVE guidelines for reporting animal research. PLoS Biol..

[18] National Research Council of the National Academies (2011). Guide for the Care and Use of Laboratory Animals.

[19] Scalbert A., Monties B., Janin G. (1989). Tannins in wood: Comparison of different estimation methods. J. Agric. Food Chem..

[20] Instituto Adolfo Lutz (IAL) (2008). Normas Analíticas.

[21] Grotto D., Bueno D.C.R., Ramos G.K., da Costa S.R., Spim S.R.V., Gerenutti M. (2016). Assessment of the safety of the shiitake culinary-medicinal mushroom, *Lentinus edodes* (*Agaricomycetes*), in Rats: Biochemical, hematological, and antioxidative parameters. Int. J. Med. Mushrooms.

[22] Gerenutti M., Del Fiol F., Groppo F. (2006). Reproductive performance of pregnant rats and embryotoxic effects of ciprofloxacin. Pharmazie.

[23] Toma A., Makonnen E., Mekonnen Y., Debella A., Adisakwattana S. (2015). Antidiabetic activities of aqueous ethanol and n-butanol fraction of Moringa stenopetala leaves in streptozotocin-induced diabetic rats. BMC Complement. Altern. Med..

[24] Volpato G.T., Damasceno D.C., Rudge M.V.C., Padovani C.R., Calderon I.M.P. (2008). Effect of *Bauhinia forficata aqueous* extract on the maternal-fetal outcome and oxidative stress biomarkers of streptozotocin-induced diabetic rats. J. Ethnopharmacol..

[25] De Mello M., de Souza C., Braga L., dos Santos J., Ribeiro I., Gobatto C. (2001). Glucose tolerance and insulin action in monosodium glutamate (MSG) obese exercise-trained rats. Physiol. Chem. Phys. Med. NMR.

[26] Aebi H. (1984). Catalase in vitro. Methods Enzym..

[27] Paglia D., Valentine W. (1967). Studies on the quantitative and qualitative characterization of erythrocyte glutathione peroxide. J. Lab. Clin. Med..

[28] Ellman G.L. (1959). Tissue sulfhydryl groups. Arch. Biochem. Biophys..

[29] Ohkawa H., Ohishi N., Yagi K. (1979). Assay for lipid peroxides in animal tissues by thiobarbituric acid reaction. Anal. Biochem..

[30] Damasceno D.C., Netto A.O., Iessi I.L., Gallego F.Q., Corvino S.B., Dallaqua B., Sinzato Y.K., Bueno A., Calderon I.M.P., Rudge M.V.C. (2014). Streptozotocin-Induced diabetes models: Pathophysiological mechanisms and fetal outcomes. Biomed. Res. Int..

[31] Lenzen S. (2008). The mechanisms of alloxan- and streptozotocin-induced diabetes. Diabetologia.

[32] Caluwaerts S., Holemans K., Van Bree R., Verhaeghe J., Van Assche F.A. (2003). Is low-dose streptozotocin in rats an adequate model for gestational diabetes mellitus?. J. Soc. Gynecol. Investig..

[33] Maritim A.C., Sanders R.A., Watkins J.B. (2003). Diabetes, oxidative stress, and antioxidants: A review. J. Biochem. Mol. Toxicol..

[34] Zhang J.J., Li Y., Zhou T., Xu D.P., Zhang P., Li S., Li H. (2016). Bin bioactivities and health benefits of mushrooms mainly from China. Molecules.

[35] Bequer L., Gómez T., Molina J.L., Álvarez A., Chaviano C., Clapés S. (2017). Experimental diabetes impairs maternal reproductive performance in pregnant Wistar rats and their offspring. Syst. Biol. Reprod. Med..

[36] Volpato G.T., Damasceno D.C., Sinzato Y.K., Ribeiro V.M., Rudge M.V.C., Calderon I.M.P. (2015). Oxidative stress status and placental implications in diabetic rats undergoing swimming exercise after embryonic implantation. Reprod. Sci..

[37] De Campos K.E., Sinzato Y.K., Pimenta W., Rudge M.V.C., Damasceno D.C. (2007). Effect of maternal obesity on diabetes development in adult rat offspring. Life Sci..

[38] Pinheiro M.S., Rodrigues L.S., Neto L.S., Moraes-Souza R.Q., Soares T.S., Américo M.F., Campos K.E., Damasceno D.C., Volpato G.T. (2017). Effect of bauhinia holophylla treatment in streptozotocin-induced diabetic rats. An. Acad. Bras. Cienc..

[39] López-Soldado I., Herrera E. (2003). Different diabetogenic response to moderate doses of streptozotocin in pregnant rats, and its long-term consequences in the offspring. Exp. Diabesity Res..

[40] Zhang Y., Mei H., Shan W., Shi L., Chang X., Zhu Y., Chen F., Han X. (2016). Lentinan protects pancreatic β cells from STZ-induced damage. J. Cell. Mol. Med..

[41] Vitak T.Y., Wasser S.P., Nevo E., Sybirna N.O. (2015). Structural changes of erythrocyte surface glycoconjugates after treatment with medicinal mushrooms. Int. J. Med. Mushrooms.

[42] Pattanathaiyanon P., Phaloprakarn C., Tangjitgamol S. (2014). Comparison of gestational diabetes mellitus rates in women with increased and normal white blood cell counts in early pregnancy. J. Obstet. Gynaecol. Res..

[43] Santilli F., Simeone P., Liani R., Davì G. (2015). Platelets and diabetes mellitus. Prostaglandins Other Lipid Mediat..

[44] Kodner C. (2016). Diagnosis and management of nephrotic syndrome in adults. Am. Fam. Physician.

[45] Eneman B., Levtchenko E., van den Heuvel B., Van Geet C., Freson K. (2015). Platelet abnormalities in nephrotic syndrome. Pediatr. Nephrol..

[46] Cho Y.I., Mooney M.P., Cho D.J. (2008). Hemorheological disorders in diabetes mellitus. J. Diabetes Sci. Technol..

[47] Klisic A., Isakovic A., Kocic G., Kavaric N., Jovanovic M., Zvrko E., Skerovic V., Ninic A. (2017). Relationship between oxidative stress, inflammation and dyslipidemia with fatty liver index in patients with type 2-diabetes mellitus. Exp. Clin. Endocrinol. Diabetes.

[48] Omodanisi E.I., Aboua Y.G., Chegou N.N., Oguntibeju O.O. (2017). Hepatoprotective, antihyperlipidemic, and anti-inflammatory activity of moringa oleifera in diabetic-induced damage in male wistar rats. Pharmacognosy Res..

[49] Green R.M., Flamm S. (2002). AGA technical review on the evaluation of liver chemistry tests. Gastroenterology.

[50] Nozaki T., Minaguchi J., Takehana K., Ueda H. (2017). Anti-Diabetic activities of traditional Chinese herbal medicine in streptozotocin-induced diabetic rats. Okajimas Folia Anat. Jpn..

[51] Akamatsu S., Watanabe A., Tamesada M., Nakamura R., Hayashi S., Kodama D., Kawase M., Yagi K. (2004). Hepatoprotective effect of extracts from lentinus edodes mycelia on dimethylnitrosamine-induced liver injury. Pharm. Soc. Japan.

[52] Rief P., Pichler M., Raggam R., Hafner F., Gerger A., Eller P., Brodmann M., Gary T. (2016). The AST/ALT (De-Ritis) ratio A novel marker for critical limb ischemia in peripheral arterial occlusive disease patients. Medicine.

[53] Afiune L.A.F., Leal-Silva T., Sinzato Y.K., Moraes-Souza R.Q., Soares T.S., Campos K.E., Fujiwara R.T., Herrera E., Damasceno D.C., Volpato G.T. (2017). Beneficial effects of *Hibiscus rosa-sinensis* L. flower aqueous extract in pregnant rats with diabetes. PLoS ONE.

[54] El-Sayyad H.I.H., Al-Haggar M.M.S., El-Ghawet H.A., Bakr I.H.M. (2014). Effect of maternal diabetes and hypercholesterolemia on fetal liver of albino Wistar rats. Nutrition.

[55] Spim S.R.V., de Oliveira B.G.C.C., Leite F.G., Gerenutti M., Grotto D. (2016). Effects of *Lentinula edodes* consumption on biochemical, hematologic and oxidative stress parameters in rats receiving high-fat diet. Eur. J. Nutr..

[56] Xu C., HaiYan Z., JianHong Z., Jing G. (2008). The pharmacological effect of polysaccharides from *Lentinus edodes* on the oxidative status and expression of VCAM-1mRNA of thoracic aorta endothelial cell in high-fat-diet rats. Carbohydr. Polym..

[57] Furukawa S., Kuroda Y., Sugiyama A. (2014). A comparison of the histological structure of the placenta in experimental animals. J. Toxicol. Pathol..

[58] Calderon I.D.M.P., Rudge M.V.C., Ramos M.D., Peraçoli J.C. (1999). Estudo longitudinal, bioquímico e histoquímico, de placentas de ratas diabéticas: Relação com a macrossomia e o retardo de crescimento intra-uterino. Rev. Bras. Ginecol. Obs..

[59] Saito F.H., Damasceno D.C., Kempinas W.G., Morceli G., Sinzato Y.K., Taylor K.N., Rudge M.V. (2010). Repercussions of mild diabetes on pregnancy in Wistar rats and on the fetal development. Diabetol. Metab. Syndr..

[60] Kc K., Shakya S., Zhang H. (2015). Gestational diabetes mellitus and macrosomia: A literature review. Ann. Nutr. Metab..

[61] Heshmat S.H. (2017). Intrauterine growth restriction. Anat. Physiol. Biochem. Int. J..

[62] Matkovics B., Kotorman M., Varga I., Hai D., Varga C. (1997). Oxidative stress in experimental diabetes induced by streptozotocin. Acta Physiol. Hung..

[63] Myatt L. (2010). Reactive oxygen and nitrogen species and functional adaptation of the placenta. Placenta.

[64] Damasceno D.C., Sinzato Y.K., Bueno A., Netto A.O., Dallaqua B., Gallego F.Q., Iessi I.L., Corvino S.B., Serrano R.G., Marini G. (2013). Mild diabetes models and their maternal-fetal repercussions. J. Diabetes Res..

[65] Lappas M., Hiden U., Desoye G., Froehlich J., Mouzon S.H., Jawerbaum A., Editors R., Eriksson U., Jain S., Levy E. (2011). The role of oxidative stress in the pathophysiology of gestational diabetes mellitus. Antioxid. Redox Signal..

[66] Patel H., Chen J., Das K.C., Kavdia M. (2013). Hyperglycemia induces differential change in oxidative stress at gene expression and functional levels in HUVEC and HMVEC. Cardiovasc. Diabetol..

[67] Santhakumari P., Prakasam A., Pugalendi K. (2003). Others modulation of oxidative stress parameters by treatment with *Piper betle* leaf in streptozotocin induced diabetic rats. Indian J. Pharmacol..

[68] Damasceno D.C., Volpato G.T., Paranhos Calderon I., Cunha Rudge M.V. (2002). Oxidative stress and diabetes in pregnant rats. Anim. Reprod. Sci..

[69] Ullah A., Khan A., Khan I. (2016). Diabetes mellitus and oxidative stress—A concise review. Saudi Pharm. J..

[70] Yurkiv B., Wasser S.P., Nevo E., Sybirna N.O. (2015). Antioxidant effects of medicinal mushrooms *Agaricus brasiliensis* and *Ganoderma lucidum* (Higher Basidiomycetes): Evidence from animal studies. Int. J. Med. Mushrooms.

